# Feasibility of mK-line and mSC-line in segments choice for laminoplasty of multilevel cervical spondylotic myelopathy

**DOI:** 10.3389/fsurg.2025.1681524

**Published:** 2025-10-30

**Authors:** Zhiming Cui, Bo Wu, Guanhua Xu, Jiajia Chen, Jinlong Zhang, Lingling Wang, Weidong Li, Hongxiang Hong, Chunshuai Wu

**Affiliations:** 1 Department of Spine Surgery, Nantong First People’s Hospital, Nantong University, Nantong, China; 2 Research Institute for Spine and Spinal Cord Disease, Nantong University, Nantong, China; 3 Key Laboratory for Restoration Mechanism and Clinical Translation of Spinal Cord Injury, Nantong, China

**Keywords:** cervical spondylotic myelopathy, laminoplasty, modified spinal cord line, modified K-line, magnetic resonance imaging

## Abstract

**Objectives:**

Laminoplasty (LAMP) is a common procedure for multilevel cervical spondylotic myelopathy (MCSM). The traditional K-line is a guide for LAMP candidate selection but is inferior to the modified K-line (mK-line) in predicting clinical outcomes. The spinal cord line (SC-line) is another indicator that considers anterior compression but is not typically used for selecting surgical segments. This study intended to propose and validate the combined application of modified spinal cord line (mSC-line) with mK-line for surgical decision-making in MCSM patients.

**Methods:**

This study included 63 MCSM patients categorized into K-line(−) group and K-line(+) group, or Type I group and Type II group based on SC-line. We defined mK-line and mSC-line in sagittal T2WI MRIs. All patients with both mK-line(+) and mSC-line(+) underwent standard LAMP. Radiographic analysis was conducted using CCI, mK-INT and mSC-INT. Clinical outcomes were evaluated by JOA, NDI and VAS scores. Preoperative and postoperative radiological outcomes and clinical outcomes were used to evaluate the prognosis and the efficacy of segmental decision-making.

**Results:**

There were no difference in baseline characteristics among all the participants. Post-operative spinal cord shift indicators (mK-INT and mSC-INT) increased significantly. The JOA score increased, while NDI and VAS scores decreased. Both the radiological outcomes and clinical outcomes demonstrated a good prognosis even in K-line(−) group and Type II group. There was a statistical correlation between JOA score recovery rate with both mK-INT and mSC-INT.

**Conclusions:**

The presence of mK-line(+) and mSC-line(+) in MRI is crucial for the selection of surgical segments in LAMP for MCSM patients. This combined criterion can help predict sufficient decompression of the cervical spinal cord and good clinical outcomes.

## 1 Introduction

Cervical spondylotic myelopathy (CSM) is a common degenerative disease that often results in progressive deterioration of nervous system (1). Surgery for moderate to severe or progressive CSM can significantly improve neurological function (2, 3). For single level or two adjacent levels, anterior decompression and fusion procedures have shown significant advantages with a lower incidence of complications (4, 5). However, multilevel cervical spondylotic myelopathy (MCSM) is often associated with spinal stenosis due to cervical disc herniation and/or ossifcation of the posterior longitudinal ligament (OPLL) (6). Posterior cervical surgery has been shown to be more effective in expanding the spinal canal with fewer associated complications, such as fusion failure, adjacent segment degeneration, internal fixation failure and dysphagia (7, 8).

Laminoplasty (LAMP) is a posterior-based procedure commonly used in the treatment of MCSM (9). LAMP provides sufficient decompression via dorsal shifting of the spinal cord from anterior compressive lesions in an expanded cervical canal (10, 11). K-line, a straight line connecting the midpoints of the anteroposterior canal diameter at C2 and C7, is considered as a useful radiological marker for predicting the prognosis of LAMP (12, 13). K-line(−) is associated with insufficient posterior decompression and unsatisfactory clinical outcomes after LAMP for MCSM patients (14). Some studies have reported that K-line(+) was a key criterion for selecting LAMP candidates, as it predicts sufficient spinal cord dorsal shift (13). However, this remains controversial because the K-line is primarily an indicator of cervical kyphosis rather than direct cord compression. Other reports proposed a modified K-line(mK-line) in magnetic resonance imaging (MRI), suggesting that an interval between the mK-line and the anterior compression factor (defined as INT) of <4 mm is associated with unsatisfactory outcomes after LAMP (15, 16). Similarly, Guangheng Xiang and colleagues proposed the spinal cord line (SC-line) on MRI to predict postoperative recovery in MCSM patients (17). They found that patients classified as Type I had the best outcomes, while Type II and Type III patients had worse outcomes. Both mK-line and SC-line in MRI attempt to evaluate the clinical outcomes based on the direct decompression of the spinal cord.

**Figure 1:**
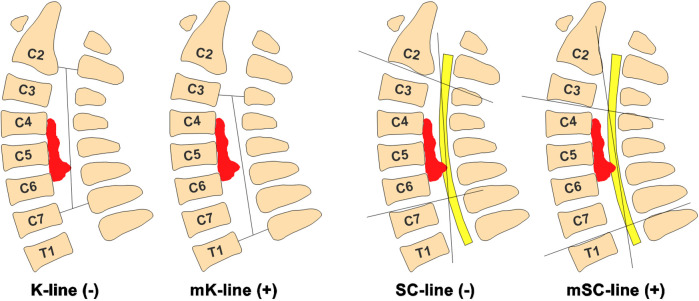
Definition of mK-line(+) and mSC-line(+) in MRI. mK-line(+): Anterior compression does not touch mK-line. mSC-line(+): Anterior compression does not touch mSC-line. On the contrary, anterior compression does touch straight line is mK-line(−) and mSC-line(−). Anterior compressive pathology (Disc herniation or OPLL).

In summary, K-line(−) patients may still be candidates for LAMP if they are mK-line(+) and have a Type I SC-line on MRI; however, the suitability of Type II and III patients remains uncertain. To our knowledge, no study has investigated the combined role of the mK-line and SC-line in surgical decision making for patients with MCSM. In this study, we defined mK-line as a straight line connecting the midpoints of the anteroposterior canal diameter at cranial and caudal vertebrae of the planned open-door segment on MRI (Figure 1). Accordingly, we propose a modified spinal cord line(mSC-line), defined as a straight line connecting the postero-inferior margin of the spinal cord at cranial and caudal vertebrae of the planned open-door segment on MRI. We simplified the classification into mSC-line(+) and mSC-line(−) (Figure 1). Thus, the mK-line and mSC-line are dynamic and vary depending on the preoperative plan for open-door segments. This study included 63 patients who underwent LAMP surgery with up to 4-year follow up, all of whom were double-positive for mK-line and mSC-line (concurrent positivity of both the mK-line and mSC-line for planned surgical segments). We aimed to investigate the combined impact of mK-line and mSC-line on surgical approach selection and their ability to predict sufficient decompression of cervical spinal cord and clinical outcome in patients after LAMP.

## 2 Materials and methods

### 2.1 Patients

This study was approved by the Ethics Committee of Nantong First People's Hospital (NO. 2025XM021). Trial registration: ClinicalTrials.gov, NCT06478485. This study collected MRI images and baseline data of 63 patients with multilevel cervical spondylotic myelopathy (MCSM) and underwent Laminoplasty (LAMP) surgery in Nantong First People's Hospital from March 2020 to March 2024 (Table 1). All participants provided informed consent. Inclusion criteria were: (1) Diagnosis of MCSM confirmed by two spine surgeons based on MRI findings and clinic signs; (2) Multi-level lesions and/or spinal stenosis (Disc herniation and OPLL) requiring LAMP; (3) Available for both preoperative and follow-up imaging data and clinical data, including sex, age, clinical symptoms, Japanese Orthopedic Association (JOA) score, Visual Analog Scale (VAS) score, Neck Disability Index (NDI) score and MRI data. The exclusion criteria were: (1) Previous cervical spine surgery; (2) Diagnosed tumor, central cord syndrome, infection or other acute traumatic injuries; (3) Diagnosed neurological disorders Parkinson's disease, polio, multiple sclerosis, or other central and peripheral nervous system diseases.

**Table 1:** Baseline characteristics of the participants.

	Values
Age (years old)	61.6 ± 8.82
Gender (male/female) number	44/19
Follow-up time (months)	22.51 ± 12.83
K-line(+)/K-line(−) number	39/24
SC-line (Type I/Type II) number	32/31
Cobb angle (degree)	−12.2238 ± 11.3684
CCI	0.1495 ± 0.1268
JOA score	13.0476 ± 3.63419
NDI score	16.2857 ± 4.31587
VAS score	5.3968 ± 1.83637

### 2.2 Radiographic analysis

**Figure 2:**
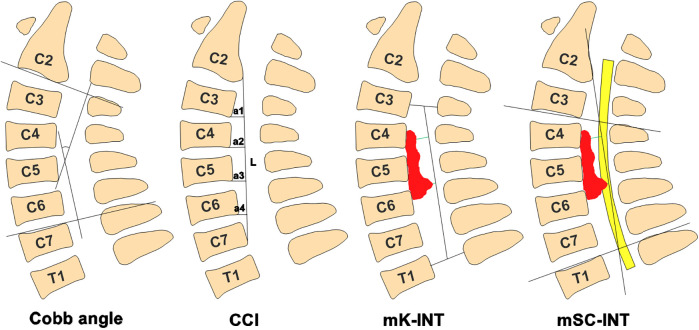
Diagram definition of Cobb angle, CCI, mK-INT and mSC-INT. The Cobb angle was measured between lines drawn along the lower endplate of C2 and the upper endplate of C7. CCI = (a1 + a2 + a3 + a4)/L*100. mK-INT, the minimum interval between the most prominent anterior compression and the mK-line; mSC-INT, the minimum interval between the most prominent anterior compression and the mSC-line.

Sagittal T2-weighted images (T2WI) were used for radiographic analysis. DICOM (Digital Imaging and Communications in Medicine) images were analyzed using RadiAnt DICOM Viewer. The Cobb angle was measured between lines drawn along the lower endplate of C2 and the upper endplate of C7. Ishihara's Cervical Curvature Index (CCI) = (a1 + a2 + a3 + a4)/L*100, where a1–4 are the distances between the posterior margins of the C3-C6 inferior endplates to a line connecting the posterior-inferior aspects of the C2 and C7 endplates, and L is the length of this line (Figure 2). The minimum interval between the most prominent anterior compression and the mK-line was defined as mK-INT; the mSC-INT was similarly defined relative to the mSC-line(Figure 2). The prefixes “pre” and “post” were used to denote preoperative and postoperative measurements, resulting in CCIpre/post, mK-INTpre/post, and mSC-INTpre/post.We have performed an inter- and intra-observer reliability analysis for the measurements of mK-INT and mSC-INT. Two experienced spine surgeons independently measured the parameters in 20 randomly selected patients twice, with a two-week interval, which showed excellent reliability (Intraclass Correlation Coefficients > 0.95 for all measurements).

### 2.3 Surgical procedure and segment selection guided by mK-line and mSC-line

Surgical segments were planned preoperatively by identifying levels where both the mK-line and mSC-line were positive. Only then was LAMP performed on those segments.

After successful general anesthesia, the patient was placed prone with the cervical spine in a slightly flexed position. A single-door cervical laminoplasty was performed using titanium miniplates. A posterior midline longitudinal incision was made, and the paraspinal muscles were dissected to expose the planned cervical spine lamina. A high-speed drill and ultrasonic bone knife were used to create the open-door side on the left and a gutter for the hinge on the right. Titanium miniplates were fixed to each lamina and the lateral mass with mini-screws to maintain the open door. Adequate decompression was confirmed by visual inspection of dural pulsations. After hemostasis, the incision was closed over a drain. A soft cervical collar was prescribed for 3 weeks postoperatively.

### 2.4 Clinical outcomes

The clinical outcomes of patients were evaluated with pre- and postoperative JOA, NDI and VAS. The time points for outcome measurements at both preoperative and final follow-up. JOA score recovery rate(%) = (postoperative score-preoperative score)/(17-preoperative score)*100. The JOA score and JOA-RR were used to measure improvement of the neurological status.

### 2.5 Statistical analysis

SPSS 20.0 (IBM) was used for statistical analysis. Pearson correlation coefcients were calculated between JOA-RR with mK-INT and mSC-INT. Data are presented as means ± standard deviations. Paired samples t-test was used to analyze the differences of both radiological outcome and clinical outcome. Pre-op vs. Post-op for all groups, *p*-value <0.05 was considered statistically meaningful.

## 3 Results

### 3.1 General overview of patients with MCSM

The data of 63 patients (44 men, 19 women) with multilevel cervical spondylotic myelopathy (MCSM), aged 61.6 ± 8.82 years, were followed up for 22.51 ± 12.83 months (Table 1). The preoperatively radiological and clinical indicators were as follows: Cobb angle was −12.2238 ± 11.3684 degree, Ishihara's Cervical Curvature Index(CCI) was 0.1495 ± 0.1268, JOA score was 13.0476 ± 3.63419, NDI score was 16.2857 ± 4.31587, VAS score was 5.3968 ± 1.83637 (Table 1). Considering the importance of K-line and SC-line for Laminoplasty (LAMP) surgery, this study divided the 63 patients into K-line(+) group and K-line(−) group, SC-line(+) group and SC-line(−) group (Table 1).

### 3.2 General radiological outcome and clinical outcome of patients with MCSM after LAMP surgery

All the patients underwent standard LAMP procedure only if the mK-line(+) and mSC-line(+) in sagittal T2-MRI image (Figure 1). This study compared the patients' radiological outcomes and clinical outcomes between pre-operation and post-operation. The data suggested that Cobb angle(degree) was −12.2238 ± 11.3684 vs. −10.3048 ± 10.78144 and CCI was 0.1495 ± 0.1268 vs. 0.1459 ± 0.1462, which is no statistical difference between pre-operation with post-operation (Table 2). The spinal cord shift indicators, including mK-INT and mSC-INT (Figure 2), were statistically larger after LAMP surgery. Likewise, clinical outcomes were significantly improved. The JOA score, an indicator of postoperative neurological status, was statistically increased (*P* < 0.01). Both the NDI score and VAS score were statistically decreased (*P* < 0.01), which indicated patients' living ability improved and pain sensation reduced respectively (Table 2).

**Table 2:** Comparison of radiological outcome and clinical outcome between pre- and postoperation.

Indicators	Pre	Post	*P*
Cobb angle (degree)	−12.2238 ± 11.3684	−10.3048 ± 10.78144	0.183
CCI	0.1495 ± 0.1268	0.1459 ± 0.1462	0.761
mK-INT (mm)	5.5075 ± 2.39138	6.4575 ± 2.55276	0.001*
mSC-INT (mm)	1.7429 ± 2.03055	2.4127 ± 2.49195	0.013*
JOA score	13.0476 ± 3.63419	15.6667 ± 2.21432	0.000*
NDI score	16.2857 ± 4.31587	5.3651 ± 5.66626	0.000*
VAS score	5.3968 ± 1.83637	1.0159 ± 1.26353	0.000*

* *P* value < 0.05.

### 3.3 Radiological outcome and clinical outcome of patients with MCSM after LAMP surgery in K-line(+) group and K-line(−) group

In order to investigate prognosis of patients with K-line(−) in MRI after LAMP surgery, we followed up these 63 patients for radiological outcome and clinical outcome. All cases met the inclusion criteria, especially defined both mK-line(+) and mSC-line(+) in preoperative MRI. The data showed that Cobb angle and CCI had no statistical difference between pre-operation with post-operation in both K-line(+) group and K-line(−) group (Table 3). The spinal cord shift indicators increased in both group after LAMP surgery, but there was no statistical difference about mSC-INT in K-line(−) group (Table 3). The clinical indicators, including JOA score, NDI score and VAS score were statistically better than pre-operation in both groups (*P* < 0.01) (Table 3).

**Table 3:** Radiological outcome and clinical outcome of K-line(−) group and K-line(+) group.

Indicators	K-line(−) group (*n* = 24)			K-line(+) group (*n* = 39)		
	Pre	Post	*P*	Pre	Post	*P*
Cobb angle (degree)	−7.9917 ± 9.52917	−7.7792 ± 7.73518	0.859	−14.8282 ± 11.73165	−11.859 ± 12.12153	0.181
CCI	0.0649 ± 0.0895	0.0727 ± 0.0965	0.49	0.2015 ± 0.1186	0.1910 ± 0.1542	0.549
mK-INT (mm)	3.9446 ± 1.71565	4.9508 ± 2.20764	0.035*	6.4692 ± 2.24876	7.3846 ± 2.31659	0.007*
mSC-INT (mm)	1.0958 ± 1.76573	1.5125 ± 2.36888	0.4	2.141 ± 2.10072	2.9667 ± 2.43076	0.01*
JOA score	14.5833 ± 1.6129	16.5833 ± 0.58359	0.000*	12.1026 ± 4.19144	15.1026 ± 2.63374	0.000*
NDI score	15.5 ± 3.31006	4.5 ± 3.95628	0.000*	16.7692 ± 4.80932	5.8974 ± 6.49208	0.000*
VAS score	5.7083 ± 1.89918	1.0417 ± 1.36666	0.000*	5.2051 ± 1.79443	1 ± 1.21395	0.000*

* *P* value < 0.05.

### 3.4 Radiological outcome and clinical outcome of patients with MCSM after LAMP surgery in SC-line gudied Type Ⅰ group and Type Ⅱ group

As we defined, mSC-line(+) means the anterior compressive lesions do not exceed the mSC line in T2-MRI, which is equivalent to including Type I (away from mSC-line) and Type II (just touched mSC-line). Previous studies have been controversial regarding the prognosis of SC-line guided Type II patients with LAMP surgery, so we investigated the radiological outcome and clinical outcome of cases in both Type I group and Type II group. Cobb angle and CCI still had no statistical difference in both Type I group and Type II group (Table 4). Both mK-INT and mSC-INT statistically increased in Type II group, but not statistically different in Type I group (Table 4). JOA score, NDI score and VAS score were also statistically better than pre-operation in both groups (*P* < 0.01) (Table 4).

**Table 4:** Radiological outcome and clinical outcome of Type I group and Type II group.

Indicators	Type I group (*n* = 32)			Type II group (*n* = 31)		
	Pre	Post	*P*	Pre	Post	*P*
Cobb angle (degree)	−16.8563 ± 12.08064	−14.2313 ± 12.06157	0.324	−7.4419 ± 8.35295	−6.2516 ± 7.51509	0.283
CCI	0.2238 ± 0.1124	0.2030 ± 0.1214	0.087	0.0727 ± 0.0906	0.0870 ± 0.1480	0.474
mK-INT (mm)	6.6897 ± 2.56409	7.4163 ± 2.83707	0.075	4.2871 ± 1.4125	5.4677 ± 1.77696	0.002*
mSC-INT (mm)	3.4313 ± 1.50535	3.8 ± 2.05002	0.323	0 ± 0	0.9806 ± 2.07821	0.013*
JOA score	12.0313 ± 4.56837	15.0625 ± 2.79328	0.000*	14.0968 ± 1.86824	16.2903 ± 1.13118	0.000*
NDI score	17.9375 ± 4.71057	6.7813 ± 6.56351	0.000*	14.5806 ± 3.1067	3.9032 ± 4.18214	0.000*
VAS score	5.6563 ± 1.7709	1.5 ± 1.29515	0.000*	5.129 ± 1.89283	0.5161 ± 1.02862	0.000*

* *P* value < 0.05.

### 3.5 Statistical correlation between neurological recorvery with postoperative mK-INT and mSC-INT in patients with MCSM after LAMP surgery

**Figure 3:**
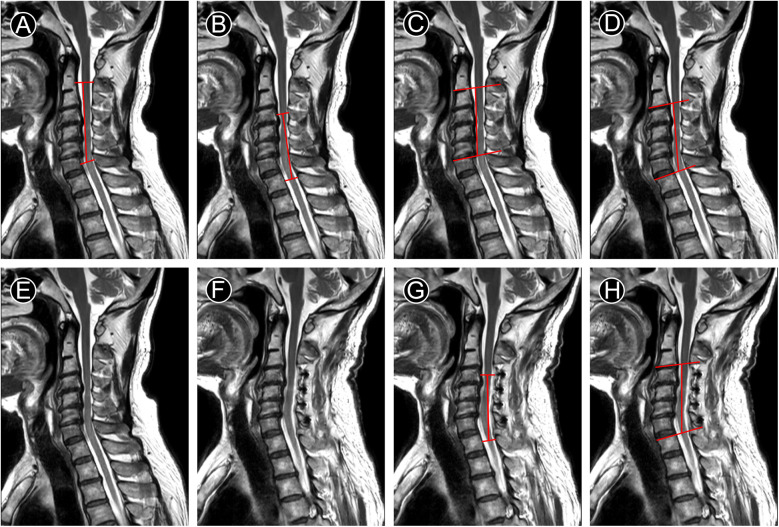
T2-MRI of a case with MCSM underwent LAMP for C4–C7. **(A–D)** preoperative measurement line. **(G–H)** postoperative measurement line. A. K-line(−), **(B)** mK-line(+), **(C)** SC-line Type III, **(D)** mSC-line(+), **(E)** preoperative image shows obstructed cerebrospinal fluid, **(F)** Image at 7 months after operation shows unobstructed cerebrospinal fluid, **(G,H)** mK-INT and mSC-INT increased.

All the data above indicated that mK-line(+) and mSC-line(+) were both important for the segments selection of LAMP surgery and good prognosis. In a case of MCSM, we modified K-line(−) and SC-line Type III to mK-line(+) and mSC-line(+) respectively (Figures 3A–D). After LAMP surgery, patient's JOA score increased from 13–17, and who was completely recovered at 7-month follow-up. Widening of the spinal canal and relief of spinal cord compression were indirectly manifested by the clearly visualization of cerebrospinal fluid on postoperative T2-MRI (Figure 3F). The spinal cord shifted backward, whose indicators mK-INT and mSC-INT increased significantly (Figures 3G,H). This study analyzed the correlation between the neurological recovery rate with postoperative radiological indicators. The data suggested JOA score recovery rate (JOA-RR) was statistically correlated with both mK-INT and mSC-INT (Table 5).

**Table 5:** Pearson correlation coefficient between JOA-RR# with postoperative mK-INT and mSC-INT.

Indicators		JOA-RR#	mK-INT	mSC-INT
JOA-RR#	Pearson coefcient	1	0.346**	0.280*
	P		0.006	0.026
	N	63	63	63
mK-INT	Pearson coefcient	0.346**	1	0.642**
	P	0.006		0.000
	N	63	63	63
mSC-INT	Pearson coefcient	0.280*	0.642**	1
	P	0.026	0.000	
	N	63	63	63

#The JOA score recovery rate (%) = (postoperative score—preoperative score)/(17—preoperative score)*100.

* *P* < 0.05.

** *P* < 0.01.

## 4 Discussion

Multilevel cervical spondylotic myelopathy (MCSM) is a common degenerative disease that can be treated with a variety of surgical procedures (18). Posterior spinal cord decompression is generally more appropriate choice for patients with a neutral or lordotic cervical spine (19). Laminoplasty approach (LAMP) has been shown to provide similar neurologic recovery to other surgical approaches, while preserving greater range of motion for the cervical spine and resulting in fewer complications (20, 21). However, patient selection for LAMP using the K-line remains controversial (14). Previous studies have reported that redefining surgical segments can convert a K-line(−) status to an mK-line(+) status (22). The feasibility of the mK-line on MRI for predicting prognosis has also been reported (23). Similarly, the SC-line classification was reported to predict outcomes, with Type I cases achieving the best recovery (17). Although both the SC-line and mK-line account for spinal cord compression, their combined use for prognostic evaluation has not been reported. This creates a dilemma for surgeons when these indicators conflict, such as in cases with mK-line (+) but SC-line Type II or III.

Our study introduces the modified spinal cord line (mSC-line) to be used in conjunction with the mK-line. Our findings demonstrate that the concurrent positivity of mK-line and mSC-line is crucial for the selection of surgical segments in LAMP. All patients in our study with this double-positive profile demonstrated significant improvements in both radiological and clinical outcomes after LAMP.

The results are particularly noteworthy for K-line(−) patients, who have historically been considered poor candidates for LAMP due to insufficient posterior decompression. Our study shows that with appropriate segment selection guided by the mK-line and mSC-line, these patients can achieve good outcomes. This indicates that the combined using of mK-line and mSC-line can provide provide a more nuanced assessment of decompression potential for spinal cord than the K-line alone.

Furthermore, our results challenge previous concerns regarding SC-line Type II patients. We found that clinical outcomes improved significantly in both Type I and Type II groups and there were some differences in radiological outcomes (such as the increase in mK-INT and mSC-INT in Type II group). This suggests that the mSC-line classification might need further refinement, and the traditional concerns about the prognosis of Type II patients after LAMP may be too restrictive. In terms of clinical practice, the use of mK-line and mSC-line in MRI can potentially provide surgeons with a more accurate and detailed guide for surgical decision-making. Surgeons can precisely determine which patients are suitable for LAMP and which segments should be included in the procedure. The combined mK-line/mSC-line assessment provides surgeons with a more precise and reliable tool for surgical planning in LAMP.

However, like any new method, there are still areas for improvement. The current method of determining mK-line and mSC-line requires experienced radiologists and spine surgeons to accurately identify the relevant anatomical points on MRI images. Developing more automated or semi-automated methods for measuring these lines could improve the reproducibility and efficiency of this approach. The development of semi-automated tools, potentially based on machine learning algorithms for spinal canal and cord segmentation, represents a critical next step. Such technology would not only enhance reproducibility across different centers and levels of expertise but also significantly improve workflow efficiency. We envision this as a essential area for future research to translate our methodological concept into a practical, user-friendly clinical application.

Secondly, our measurements are based on supine MRI, which does not capture the standing alignment and axial loading of the spine. The discrepancy between supine and standing alignment could theoretically affect the accuracy of our predictions for postoperative cord shift. Future studies incorporating standing MRI or weight-bearing CT could address this limitation.

Our future research would focus on external validation in a larger, multi-center cohort to confirm the generalizability of our findings. Furthermore, while our correlation analysis demonstrates a significant association between radiographic parameters and clinical recovery, the sample size limited our ability to build a robust multivariate predictive model that controls for all potential confounders. Future studies with larger cohorts are warranted to develop and validate such predictive models. Additionally, exploring the long-term effects of using mK-line and mSC-line guided LAMP, such as the development of adjacent segment degeneration over time, is also essential. Understanding the long-term impact of this new approach will help to further optimize the treatment of MCSM patients.

## 5 Conclusions

The mK-line(+) and mSC-line(+) in MRI could be used for the selection of surgical segments in LAMP for sufficient decompression of the cervical spinal cord and good clinical outcomes in MCSM patients.

## Data Availability

The original contributions presented in the study are included in the article, further inquiries can be directed to the corresponding author.
